# Education mitigates age-related decline in N-Acetylaspartate levels

**DOI:** 10.1002/brb3.311

**Published:** 2015-01-29

**Authors:** Kirk I Erickson, Regina L Leckie, Andrea M Weinstein, Polina Radchenkova, Bradley P Sutton, Ruchika Shaurya Prakash, Michelle W Voss, Laura Chaddock-Heyman, Edward McAuley, Arthur F Kramer

**Affiliations:** 1 Department of Psychology, University of PittsburghPittsburgh, Pennsylvania; 2 Center for the Neural Basis of Cognition, University of PittsburghPittsburgh, Pennsylvania; 3 Center for Neuroscience, University of PittsburghPittsburgh, Pennsylvania; 4 Department of Bioengineering, University of IllinoisUrbana, Illinois; 5 Beckman Institute for Advanced Science and Technology, University of IllinoisUrbana, Illinois; 6 Department of Psychology, Ohio State UniversityColumbus, Ohio; 7 Department of Psychology, University of IowaIowa City, Iowa; 8 Department of Psychology, University of IllinoisUrbana, Illinois; 9 Department of Kinesiology and Community Health, University of IllinoisUrbana, Illinois

**Keywords:** Aging, brain reserve, cognitive reserve, education, fitness

## Abstract

**Background:**

Greater educational attainment is associated with better neurocognitive health in older adults and is thought to reflect a measure of cognitive reserve. In vivo neuroimaging tools have begun to identify the brain systems and networks potentially responsible for reserve.

**Methods:**

We examined the relationship between education, a commonly used proxy for cognitive reserve, and N-acetylaspartate (NAA) in neurologically healthy older adults (*N* = 135; mean age = 66 years). Using single voxel MR spectroscopy, we predicted that higher levels of education would moderate an age-related decline in NAA in the frontal cortex.

**Results:**

After controlling for the variance associated with cardiorespiratory fitness, sex, annual income, and creatine levels, there were no significant main effects of education (B = 0.016, *P* = 0.787) or age (B = −0.058, *P* = 0.204) on NAA levels. However, consistent with our predictions, there was a significant education X age interaction such that more years of education offset an age-related decline in NAA (B = 0.025, *P* = 0.031). When examining working memory via the backwards digit span task, longer span length was associated with greater education (*P* < 0.01) and showed a trend with greater NAA concentrations (*P* < 0.06); however, there was no age X education interaction on digit span performance nor a significant moderated mediation effect between age, education, and NAA on digit span performance.

**Conclusions:**

Taken together, these results suggest that higher levels of education may attenuate an age-related reduction in neuronal viability in the frontal cortex.

## Introduction

Higher levels of education are consistently associated with a reduced risk for cognitive impairment (Stern et al. 1994; Stern 2002; Yaffe et al. 2009; Barnes and Yaffe 2011; Sharp and Gatz 2011). This association is thought to provide evidence for the cognitive reserve hypothesis, which proposes that cognitive stimulation (often measured by proxies for stimulation, including higher levels of education) helps to maintain superior levels of cognitive function even in the face of age-related brain pathology (Stern 2009, 2012). A related concept, brain reserve, is usually defined as a quantifiable measure of neuronal integrity or mass that is also related to cognitive stimulation or education and may be an antecedent to cognitive reserve (Stern 2009). Thus, one distinction between cognitive and brain reserve rests on the outcome measure: cognitive reserve is linked to a functional outcome or behavior (e.g., cognitive performance), whereas brain reserve is linked to neural mass, size, volume, or count (e.g., number of neurons). Therefore, the two concepts are closely linked but not identical. Greater brain reserve may elicit more flexible brain circuits allowing for the compensation of other losses, thereby providing the capacity for greater cognitive reserve. On the other hand, cognitive reserve may not always be dependent on brain reserve. That is, the integrity and plasticity of brain circuitry during cognitive challenges may be independent of the mass, size, or the number of neurons composing the region.

Although many studies have reported that greater amounts of education are related to several in vivo measures of brain function and brain health including greater gray matter volume (Sole-Padulles et al. 2009; Murray et al. 2011; Arenaza-Urquijo et al. 2013a,b), connectivity (Arenaza-Urquijo et al. 2013a), task-evoked brain activation (Sole-Padulles et al. 2009), and white matter microstructure (Teipel et al. 2009; Piras et al. 2011), it remains unclear which of these measures are more proximal to the concepts of cognitive or brain reserve. More specifically, as cognitive reserve focuses on functional outcomes and individual differences in task processing, task-evoked patterns of brain activation using functional magnetic resonance imaging (fMRI) might be the most proximal marker to cognitive reserve, whereas measures of structural integrity, cortical thickness, or volume might be more proximal to brain reserve. Thus, neuroimaging metrics may lie along a gradient with some measures (e.g., cortical thickness) more conceptually linked to brain reserve and other measures (e.g., task-evoked fMRI) more closely linked to cognitive reserve (Stern 2009).

One challenge with this view is that we still have a very poor understanding of what structural and volumetric brain measures reflect on a cellular level, thus making it difficult to interpret morphological measures of the brain as unequivocal markers of brain reserve. Hence, it is important to establish methods that might be more directly associated with neuronal integrity, and thus, brain reserve. For example, magnetic resonance spectroscopy can be used to examine metabolites that are considered to be measures of neuronal viability. N-acetylaspartate (NAA) is a nervous system-specific metabolite found almost exclusively in cell bodies of neurons where it plays a critical role in cellular metabolism and myelination (Nadler and Cooper 1972; Moffett et al. 1991). NAA is essential for normal brain operation and declines in several neurodegenerative and neuropsychiatric diseases including Alzheimer's Disease, multiple sclerosis, and substance abuse disorder (Moffett et al. 2007). In contrast, there are minimal changes in NAA concentrations in normal aging (Wu et al. 2012) indicating that NAA may be a viable marker to distinguish normal aging from early dementia (Reyngoudt et al. 2012). Because of its exclusivity to neurons, NAA is considered an in vivo marker of neuronal viability and metabolism, and therefore may be a marker for brain reserve. We predicted that higher educational levels would be associated with higher NAA levels and offset an age-related decline in NAA concentrations in a group of healthy, nondemented, older adults.

## Methods

### Participants

Participants (*N* = 135, 90 females) between the age of 59 and 80 (M = 65.97) were recruited from Champaign–Urbana and east-central Illinois for a 1-year-randomized exercise intervention. The Modified Mini-Mental Status Examination (Stern et al. 1987) was used to screen for cognitive impairment and scores <51 (a high score of 57) were used as criteria for exclusion. Inclusion criteria included normal or corrected to normal vision and absence of clinical depression (assessed by the five-item Geriatric Depression Scale [>3; (Sheikh and Yesavage 1986)]. None of the participants had a history of head trauma, head or neck surgery, neuropsychiatric or neurological conditions, diabetes, or ferrous metallic implants. Self-reported use of psychiatric or neurological medications was also used as exclusionary criteria. The results reported here were from the baseline assessment of brain health and function collected as part of the randomized intervention. All participants signed an informed consent approved by the University of Illinois and provided physician's consent to engage in fitness testing.

### Magnetic Resonance Spectroscopy (MRS) protocol and data processing

**Figure 1:**
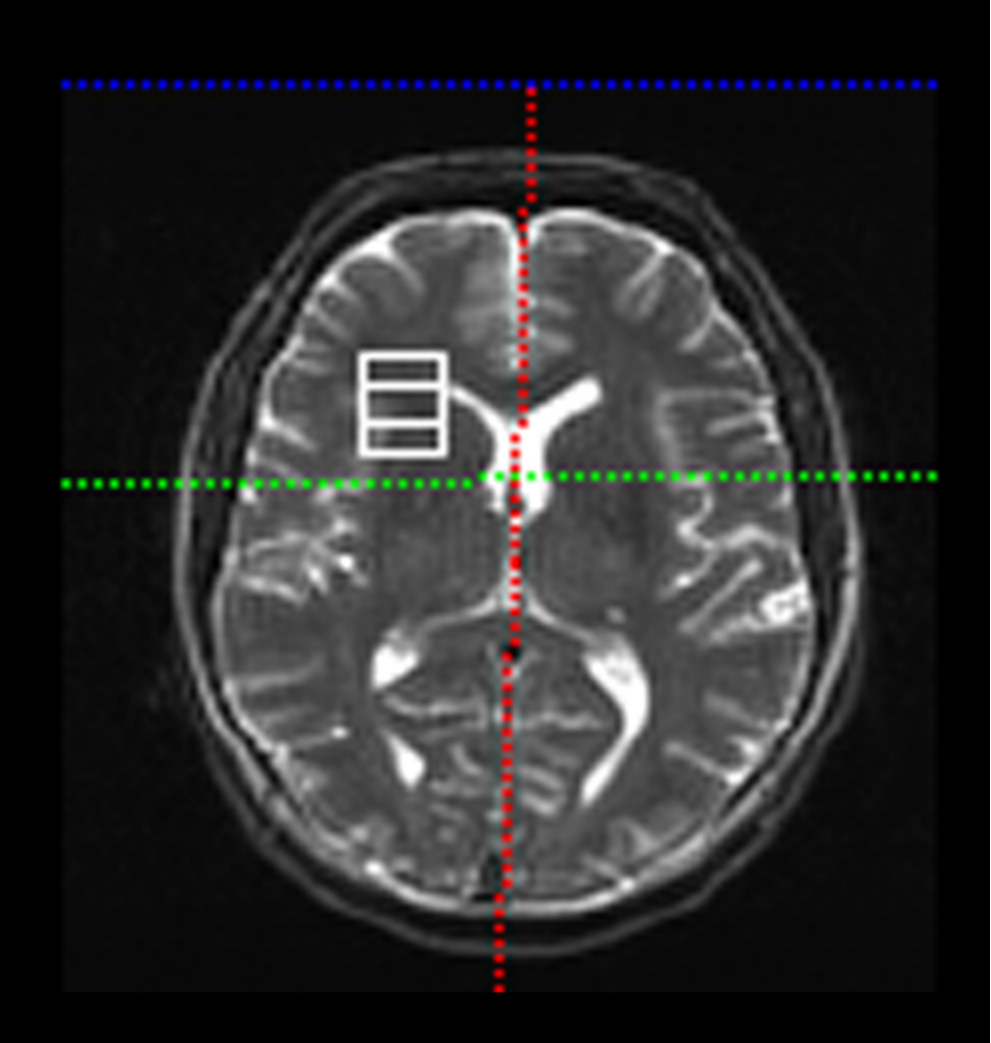
Location of the single voxel in the magnetic resonance spectroscopy sequence. The voxel contained portions of the inferior frontal gyrus, middle frontal gyrus, insula, and anterior basal ganglia.

A Siemens 3 Tesla Allegra was used for MRS acquisition. A spin-echo single-voxel spectroscopy sequence was used for the acquisition of a single 18-mm isotropic voxel in the right frontal cortex [Repetition Time (TR)=2000 msec and Echo Time (TE) = 30 msec]. The single voxel included gray and white matter tissue, but did not include CSF. Its position encompassed the inferior frontal gyrus, insula, and anterior portions of the basal ganglia (Fig.1). The acquisition used water saturation and 128 averages of the spectroscopy acquisition with a 1200-Hz bandwidth. The data were processed in the Siemens Syngo 2004 spectroscopy analysis package (80333 Munich, Germany), which provides peak fitting of metabolites in proton spectroscopy. NAA and Creatine (Cr) levels were extracted from the data and used for analysis.

### Age, education, fitness, and socioeconomic variables

Self-reported age and years of education were recorded and used as primary variables of interest. Aerobic fitness (VO_2_ peak) was assessed by graded maximal exercise testing on a motor-driven treadmill and used as a covariate [details provided in (Erickson et al. 2012)], see Statistical analysis section for details. As socioeconomic status (SES) is linked to years of education, we used a proxy for SES, annual income, divided into eight categories, as a covariate in the statistical model (coded categorically as follows: <$5000; $5001–$10,000; $10,001–$15,000; $15,001–$20,000; $20,001–$25,000; $25,001–$30,000; $30,001–$40,000; >$40,000) to isolate the relationship between education, rather than income, on NAA. Gender was also used as a statistical covariate. See Table 1.

**Table 1:** Sample Demographics and correlations between variables of interest

	Mean	SD	Sex	Age	Cri	Inc	NAA	Education
Sex (% female)	65.3		1					
Age	65.968	5.313	0.025	1				
Cri	8.692	1.445	−0.317**	−0.157	1			
Income (% <$40,000)	41.9%		0.143	−0.144	0.058	1		
Income Category (%*N*)								
<$5000	1.6							
$5001–$10,000	0.8							
$10,001–$15,000	3.2							
$15,001–$20,000	5.6							
$20,001–$25,000	4.8							
$25,001–$30,000	6.5							
$30,001–$40,000	19.4							
>$40,000	58.1							
NAA	15.005	2.449	−0.369**	−0.207*	0.504**	0.047	1	
Education (years)	15.718	2.834	0.246**	−0.056	−0.083	0.174	0.009	1
Age × Education			−0.178*	0.035	0.042	0.151	0.181*	−0.008

* *P* < 0.05.

** *P* < 0.01.

### Cognitive assessment

A comprehensive neuropsychological battery was administered to participants. Given that the MRS voxel was placed in the prefrontal cortex we used a measure of working memory from the Digit Span subtest of the Wechsler Adult Intelligence Scale Third Edition (Wechsler, 1997) as the primary outcome variable of interest because the prefrontal cortex supports working memory function and we have found previously that the MRS data are associated with digit span performance (Erickson et al. 2012). The digit span test was collected on all participants approximately 2 weeks before the MRS session. In this test, participants were read aloud a list of numbers at an interval of 1 digit per second, starting with two digits and adding one digit after two correct responses. In the forward span condition, the participant was instructed to orally repeat the list of numbers verbatim without writing anything down. In the backward span condition, participants were asked to repeat the list of numbers in reverse order of presentation. The length of the to-be-remembered numbers increased until the participant incorrectly responded to two presentations of the same length in a row or until the maximum span length was reached. The outcome measure of this test was span length, or the greatest number of digits correctly repeated (range 0–9 digits for forward span; 0–8 digits for backward span).

### Statistical analysis

All variables except for annual income conformed to assumptions of normality. A log-transformation on the income variable did not correct the skew, so the original untransformed variable was retained for analysis. We began by conducting bivariate Pearson correlations to assess the relationships between variables of interest. Multiple Regression was used to determine the association between Age, Education, and the interaction between Age and Education on NAA concentrations. To determine main effects, covariates including sex, annual income, creatine (Cr), and VO_2_ peak, were entered into the regression model, followed by Age and Education. We did not examine NAA in relation to Cr (NAA:Cr) because any significant association could be driven by variation in either the numerator or denominator of the ratio even though only the numerator (NAA) was of interest. Hence, Cr was entered as a covariate rather than as a ratio to NAA. The interaction term (Age x Education) was entered last in the model to examine whether greater amounts of education moderated an age-related change in NAA:


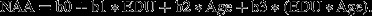


The resampling technique of bootstrapping was employed, using 1000 permutations of random sampling with replacement to determine the conditional effect of education on the association between age and NAA. Bootstrapping is the statistical method of random resampling, with replacement, from the sample distribution to create an approximate comparison distribution. This approximate distribution is used for hypothesis testing, rather than testing against a known distribution (e.g., *z*-distribution, *t*-distribution). The original sample data are then compared to this resampled distribution, and a bootstrap estimate is derived from where the data lies on the bootstrap sampling distribution. These distributions are nonparametric, and the *P*-values derived from the bootstrap distributions are also nonparametric. A threshold of *P* < 0.05 was used for all comparisons. Bias-corrected and accelerated confidence intervals were used for hypothesis testing, meaning that the confidence intervals adjusted for skewness and any over- or under-estimation of the population parameter. Please see Hayes (2013) for further details on bootstrapping.

The Johnson–Neyman technique [originally described in (Johnson and Neyman 1936)] was applied to the conditional model in order to determine a threshold of significance. The Johnson–Neyman technique is an analysis of covariance (ANCOVA) where the relationship between age and NAA levels are assumed to be linear but nonparallel at varying degrees of the moderator, education. A region of significance is then identified by applying fixed values of years of education, across the range of sample data, to the regression equation. This technique was applied using the PROCESS program, developed by Andrew Hayes (Hayes 2013).

## Results

### Correlations

N-acetylaspartate was significantly correlated with age (*r* = −0.207, *P* = 0.016) and sex (*r* = −0.369, *P* = 0.001), where males and younger individuals displayed higher NAA concentrations. We found a significant association between fitness and education (*r* = 0.278, *P* = 0.001), such that individuals with higher education had a higher VO_2_ peak. There was also a significant association between education and sex, such that females had more years of schooling (*r* = 0.246, *P* = 0.004). Hence, sex, annual income, Cr, and VO_2_ peak were entered as covariates in the multiple regression analysis. See Table 1.

### Main effects of age and education

Inconsistent with our hypotheses, we did not find a significant main effect of education (B = 0.016, *P* = 0.787) or age (B = −0.058, *P* = 0.204) on NAA levels after controlling for sex, fitness, annual income, and Cr in the multiple regression model.

### Education moderates effect of age on NAA

**Figure 2:**
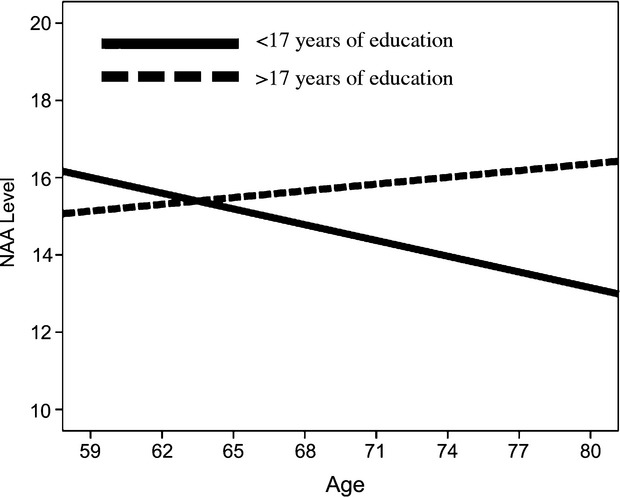
For graphical purposes, the sample was divided into groups, fewer years of education (<17 years; solid line) and higher amounts of education (>17 years; dotted line), based on the Johnson–Neyman calculated threshold of significance. The figure shows that older age is associated with lower NAA levels, but only in the lower education group. This effect was significant after controlling for sex, fitness level, Cr, and income.

Despite no significant main effects of education or age, a significant interaction was revealed between age and education on NAA levels (B = 0.025, *P* = 0.031) such that more years of education offset an age-related difference in NAA (see Fig.2; Table 2). The Johnson–Neyman (JN) technique was used to determine the point of transition at which the number of years of education is sufficient for detecting an age-related difference in NAA levels (Johnson and Fay 1950; Preacher and Hayes 2008). The JN values illustrate that the relationship between age and NAA levels is conditional on years of education in individuals with less than or equal to 17.09 years of education (B = −0.087, *P* = 0.05). This is true for 71.77% of the sample. Education did not significantly moderate the effect of age on NAA levels in individuals with more than 17.09 years of education, accounting for 28.23% of the sample (See Table 3).

**Table 2:** Moderation analysis coefficients. Bootstrapping is the statistical method of random resampling from the sample distribution, with replacement, to create an approximate sample distribution. This approximate distribution is used for hypothesis testing, rather than testing against a known distribution (e.g., *z*-distribution). The original sample data is then compared to this sample distribution. Bias is the tendency of a sample statistic to over- or under-estimate the population parameter. Bias correction in the confidence intervals of a bootstrap estimate attempt to minimize the bias in the statistic by adjusting the confidence intervals based on the expectation values. A bias-corrected and accelerated confidence interval further adjusts for skewness in the bootstrap distribution. These distributions are nonparametric, and the p-values derived from the bootstrap distributions are also nonparametric. See Table 3 for details of the confidence intervals

Bootstrap Coefficients	B	Bias	*P*
Income	−0.019	0.012	0.002
Sex	−1.660	−0.006	0.891
Cr	0.594	0.001	0.001
VO_2_	0.127	−0.003	0.001
Education	0.016	0.002	0.788
Age	−0.058	0.000	0.192
Interaction (Age × Education)	0.025	0.000	0.031

**Table 3:** Conditional effect of Age on NAA Levels at values of Years of Education

Education (years)	Effect	Standard error	*t*	*P*	LLCI	ULCI
8	−0.2451	0.1236	−1.9825	0.0497	−0.4899	−0.0003
8.8	−0.2311	0.1122	−2.0603	0.0416	−0.4533	−0.009
9.6	−0.2172	0.1009	−2.1524	0.0334	−0.417	−0.0174
10.4	−0.2033	0.0899	−2.2622	0.0255	−0.3812	−0.0253
11.2	−0.1893	0.0791	−2.3934	0.0183	−0.346	−0.0327
12	−0.1754	0.0688	−2.549	0.0121	−0.3116	−0.0391
12.8	−0.1615	0.0592	−2.7273	0.0074	−0.2787	−0.0442
13.6	−0.1475	0.0507	−2.911	0.0043	−0.2479	−0.0472
14.4	−0.1336	0.0439	−3.0449	0.0029	−0.2205	−0.0467
15.2	−0.1196	0.0397	−3.0153	0.0031	−0.1982	−0.0411
16	−0.1057	0.039	−2.7137	0.0076	−0.1829	−0.0286
16.8	−0.0918	0.0419	−2.1916	0.0304	−0.1747	−0.0089
17.0946	−0.0866	0.0438	−1.9803	0.05	−0.1733	0
17.6	−0.0778	0.0478	−1.6292	0.1059	−0.1725	0.0168
18.4	−0.0639	0.0557	−1.1468	0.2538	−0.1743	0.0464
19.2	−0.05	0.065	−0.7691	0.4434	−0.1786	0.0787
20	−0.036	0.075	−0.4802	0.632	−0.1846	0.1125
20.8	−0.0221	0.0856	−0.2581	0.7968	−0.1917	0.1475
21.6	−0.0082	0.0966	−0.0845	0.9328	−0.1994	0.1831
22.4	0.0058	0.1078	0.0536	0.9574	−0.2076	0.2192
23.2	0.0197	0.1191	0.1654	0.8689	−0.2162	0.2557
24	0.0336	0.1307	0.2575	0.7972	−0.2251	0.2924

**Note**: Moderator value(s) defining Johnson–Neyman significance region.

### NAA and working memory

Our previous work has shown that NAA mediates a relationship between fitness and backwards digit span performance in older adults (Erickson et al. 2012). On the basis of this finding, we examined whether NAA levels would predict backwards digit span performance. We found a trend for NAA to predict backwards digit span length when controlling for income, sex, Cr, and fitness (B = −0.117, *P* = 0.06). However, there was a significant association between years of education and span length (B = 0.138, *P* < .01), such that individuals with more years of education had a greater digit span score. In contrast, we failed to find either a significant association between age and span length, or a significant Age x Education interaction on span length (all *P*'s > 0.30). Using the Preacher–Hayes PROCESS toolbox (http://www.afhayes.com/spss-sas-and-mplus-macros-and-code.html), we modeled the moderation–mediation pathway to test whether education moderates the mediating effect of NAA on the association between age and digit span performance. No significant moderated mediation model was found for either the forwards (B = 0.0004, SE = 0.0012, −0.0015 ± 0.0036) or backwards digit span tasks (B = −0.0015, SE = 0.0018, −00069 ± 0.0006).

## Discussion

We predicted that more years of education would offset age-related differences in frontal cortex NAA, consistent with the hypotheses of brain reserve. Consistent with our prediction, we found a significant Age x Education interaction revealing that higher levels of education offset an age-related decrease in NAA. This effect was significant even with including annual income, Cr, fitness levels, and sex as covariates. These results are especially important given that several studies have found nonsignificant declines in NAA in normal aging [e.g., (Wu et al. 2012)], suggesting that NAA may be a viable marker to distinguish normal aging from pathological aging. Our results extend these findings and suggest that unexplored variance associated with years of education may be an important factor concomitantly modifying normal age-related decline in NAA and risk for dementia.

Our results support current evidence that more years of education may be protective against brain dysfunction and impairment in late life. Higher education is often conceptualized as a measure for cognitive reserve as it is assumed that more years of education provides opportunities for greater intellectual stimulation throughout the lifespan, which in turn increases the capacity to utilize alternative strategies or compensatory approaches to perform better in the face of pathology. In contrast, brain reserve is conceptually similar to cognitive reserve, but is related to neuronal count or mass rather than a functional outcome. Higher levels of education are also associated with in vitro markers of brain reserve including gray matter density (Arenaza-Urquijo et al. 2013a,b). From this perspective, we reasoned that NAA could be an in vivo marker for brain reserve given its link to neuronal integrity.

An alternative explanation for our results is that more years of education is a marker of an elevated socioeconomic condition, a marker for better access to health care services, or a marker for a heighted awareness of the importance of healthy behaviors and lifestyles. To partially account for several of these alternative explanations, we included annual income and cardiorespiratory fitness levels as covariates in the statistical model and the moderating effect of education on age-related NAA patterns remained significant. However, annual income is only one marker of SES, and cardiorespiratory fitness is only one measure of overall physical health. Hence, we cannot rule out the possibility that our results may support an SES or physical health hypothesis rather than a brain reserve hypothesis. More sophisticated measures, and/or aggregate measures of socioeconomic, physical health, and education are needed to make distinctions among these associations.

Nonetheless, our results are consistent with prior literatures linking neuroimaging outcomes with years of education. This body of research has reported that more years of education are often associated with elevated brain health and function [see (Bartres-Faz and Arenaza-Urquijo 2011)]. Here, we proposed to study this association using a more direct measure of neuronal viability, NAA. NAA is a metabolite found almost exclusively in cell bodies of neurons where it is involved in cellular metabolism (Moffett et al. 2007). Evidence for the importance of NAA comes from Canavan disease, an autosomal-recessive neurodegenerative mutation that deacetylates NAA, causing severe cognitive and psychomotor deficits and usually resulting in death before 18 months of age (Matalon et al. 1988). Furthermore, reduced NAA or NAA:Cr concentrations have been reported in Alzheimer's disease, stroke, multiple sclerosis, epilepsy, schizophrenia, and substance abuse disorder [see reviews (Moffett et al. 2007; Ross and Sachdev 2004)].

The results reported here are consistent with a prior study in older adults (*N* = 97) reporting that higher levels of education were associated with elevated levels of whole brain NAA concentrations (Glodzik et al., 2012). Glodzik and colleagues reported that the positive association between education and whole brain NAA were only apparent in those aged 51–70 years, whereas those aged 71–89 years did not show an association between education and NAA. In contrast, we found that the association between education and NAA was greater at older ages (>68 years) compared to younger ages (<68 years). This difference between studies may be explained by the greater age-range in the Glodzik et al., study. Furthermore, Glodzik et al. (2012) did not control for fitness levels or annual income in their analyses. Hence, it will be important for future studies to follow up with additional age groups and metrics of health and socioeconomic advantage to test the effect moderation reported by both Glodzik et al. (2012) and the results we report here.

Despite the significant Age x Education interaction on NAA levels, we failed to find a similar association with the digit span task. Hence, it is difficult to interpret the higher NAA concentrations in the frontal cortex of educated older adults in relation to cognitive performance. Future studies with both a broader array of working memory tests and a denser sampling of NAA throughout the brain may help clarify the association between education-related associations with NAA and cognitive function.

There are several limitations to this study. First, we utilized a measure that captured the quantity of education, but it is likely that *quality* of education also plays an important role on cognitive development and decline in late life that is independent from years of education (Manly et al. 2005). For example, children graduating from less affluent school districts may not have been exposed to the same opportunities to excel as children from more affluent districts. Thus, the quantity, or number of years of education, may be significantly different from measures of the quality of the schooling. Along these lines, more sophisticated measurements of education, socioeconomic advantage, and relevant health behaviors could help to interpret and expand on the results reported here (Stern et al. 2005). Second, the majority of our sample had some college education and do not accurately reflect the population as a whole, so our effects may be a conservative estimate of the effect of education on mitigating age-related losses in NAA. It will be important for future studies to examine a sample with a more varied educational background. Third, although we examined NAA in relation to digit span performance, this is only one measurement of working memory functioning. Future studies that utilize more sensitive working memory measures should examine whether NAA explains variability in performance. Fourth, even though all participants were carefully screened for psychiatric and neurological conditions, it is possible that preclinical neuropathology was affecting neurocognitive health. Finally, scanner limitations forced us to examine NAA levels in a single voxel. We decided to focus on NAA concentrations in the frontal cortex, as the frontal cortex is especially susceptible to age-related neural changes. Future studies that can capitalize on the recent developments in MR spectroscopy that allow for multiple voxel acquisition can overcome this limitation. Our group, and others, should use multivoxel spectroscopy methods, with a more heterogeneous sample to explore effects of SES, quality of education, and other factors that may covary, and explain, some of the associations we describe here. Furthermore, future studies could examine how education interacts with other promising approaches (i.e., physical activity) for enhancing cognitive function throughout the lifespan.

In sum, we find that higher levels of education offset an age-related decline in NAA levels in the frontal cortex. As NAA is an in vivo marker of neuronal viability, our results suggest that more years of education may prevent neuronal loss in late life and that NAA may be a useful marker for assessing brain reserve, health behaviors, or socioeconomic advantages in relation to brain integrity.
